# Sex differences in the morphological failure patterns following hip resurfacing arthroplasty

**DOI:** 10.1186/1741-7015-9-113

**Published:** 2011-10-13

**Authors:** Andrea Hinsch, Eik Vettorazzi, Michael M Morlock, Wolfgang Rüther, Michael Amling, Jozef Zustin

**Affiliations:** 1 Institute of Pathology, University Medical Center Hamburg-Eppendorf, Germany; 2 Department of Medical Biometry and Epidemiology, University Medical Center Hamburg-Eppendorf, Germany; 3 Biomechanics Section, TUHH University of Technology Hamburg-Harburg, Germany; 4 Department of Orthopaedics, University Medical Center Hamburg-Eppendorf, Germany; 5 Institute of Osteology and Biomechanics, University Medical Center Hamburg-Eppendorf, Germany

## Abstract

**Background:**

Metal-on-metal hybrid hip resurfacing arthroplasty (with a cementless acetabular component and a cemented femoral component) is offered as an alternative to traditional total hip arthroplasty for the young and active adult with advanced osteoarthritis. Although it has been suggested that women are less appropriate candidates for metal-on-metal arthroplasty, the mechanisms of prosthesis failure has not been fully explained. While specific failure patterns, particularly osteonecrosis and delayed type hypersensitivity reactions have been suggested to be specifically linked to the sex of the patient, we wished to examine the potential influence of sex, clinical diagnosis, age of the patient and the size of the femoral component on morphological failure patterns in a large cohort of retrieved specimens following aseptic failure of hip resurfacing arthroplasty.

**Methods:**

Femoral remnants retrieved from 173 hips with known patient's sex were morphologically analyzed for the cause of failure. The results were compared with the control group of the remaining 31 failures from patients of unknown sex. The odds ratios (OR) and 95% confidence intervals (CI) of the following morphologically defined variables were calculated using logistic regression analysis: periprosthetic fractures (n = 133), osteonecrosis (n = 151), the presence of excessive intraosseous lymphocyte infiltration (n = 11), and interface hyperosteoidosis (n = 30). Logistic regression analysis was performed both unadjusted and after adjustment for sex, age, the size of the femoral component, and preoperative clinical diagnosis.

**Results:**

Femoral remnants from female patients had a smaller OR for fracture (adjusted OR: 0.29, 95% CI 0.11, 0.80, *P *for difference = 0.02) and for the presence of osteonecrosis (adjusted OR: 0.16, 95% CI 0.04, 0.63, *P *for difference = 0.01). However, women had a higher OR for both the presence of excessive intraosseous lymphocyte infiltration (adjusted OR: 10.22, 95% CI 0.79, 132.57, *P *for difference = 0.08) and interface hyperosteoidosis (adjusted OR: 4.19, 95% CI 1.14, 15.38, *P *for difference = 0.03).

**Conclusions:**

Within the limitations of this study, we demonstrated substantial sex differences in distinct failure patterns of metal-on-metal hip resurfacing. Recognition of pathogenically distinct failure modes will enable further stratification of risk factors for certain failure mechanisms and thus affect future therapeutic options for selected patient groups.

## Background

Gender medicine is a novel and rapidly evolving research discipline. Indeed, there has been an almost linear increase in the literature incorporating sex/gender differences [1]. Within the last few years, lively discussion regarding possible sex differences has also been initiated in the orthopedic surgeon community. Serious concerns have arisen regarding the potential adverse biological reactions to metal-bearing surfaces and particular prosthesis designs such as hip resurfacing arthroplasty. In fact, metal-on-metal technology is now used in over one-third of all hip arthroplasties performed in the United States [2]. In recent years, hip resurfacing arthroplasty has become an accepted alternative to traditional stemmed total hip arthroplasty in young adults worldwide [3], although patient selection is important in order to avoid failure [4-11]. Most authors [2-6,8-12] consider men under the age of 65 with osteoarthritis to be the best candidates for hip resurfacing. However, recent reports from centers that design hip resurfacing arthroplasty [7,13] suggest that the smaller size of the femoral component rather than female sex is linked with worse outcomes for this procedure.

In our earlier studies on failed hip resurfacing arthroplasty, we observed some sex differences in a large collection of retrieved prostheses: men were more frequently revised for postnecrotic fractures [14], and the extent of osteonecrosis was larger than in specimens obtained from women [14]. However, women were more frequently revised for unexplained persistent groin pain, which was attributed to a suggested hypersensitivity reaction after the index surgery [15]. In the present study, we calculated the ORs for morphologic failure modes in the entire cohort after adjustment for sex, age, the size of the femoral component, and preoperative clinical diagnosis. We asked: is the previously reported sex dimorphism really linked with the sex of the patient?

## Methods

### Data collection

In an international multi-surgeon retrieval study on total hip resurfacing arthroplasty (THRA), we obtained 283 specimens between January 2004 and February 2010. During the planning of the design of this study in 2003, the suggested primary objective was a tribological investigation of the prosthesis surface in order to demonstrate the potential wear-induced failures as they were frequently reported in the second generation of (metal-on-polyethylene) THRA. Therefore, several specimens, preferentially from the early phase of the Hamburg retrieval study on THRA were obtained without bone tissues or without using any standard fixation method for bone tissue (Table 1). Later on, when we presented preliminary results of morphological analyses of the first dozen standard analyzed retrieved hips and specifically focused on the issue of histopathological changes within the periprosthetic tissues and the potential adverse reactions to metal material, the discipline of the cooperating surgeons in the submission of basic clinical data substantially improved. Altogether, 46 specimens did not contain bone remnant tissues under the cup at all; in 16 cases focal rests (mostly less than 2 cm^2^) of the bone tissue were severely mechanically damaged and 11 specimens contained osseous tissue but were sent without fixation and the histopathology was non-informative. We also obtained 31 cases with minimal clinical data; particularly the data on sex were completely missing. Finally, six cases were revised for periprosthetic infections and were not included in further analyses. After excluding all 79 cases with septic complications, insufficient quality of fixation of the femoral remnant bone tissue and hips with invalid demographic data, the present study cohort contained 85 women (median age 56 years old, interquartile range (IQR) 49 to 60) and 88 men (median age 56 years old, IQR 51 to 60; *P *= 0.584; Table 2). Valid clinical data were obtained for the majority of the specimens in the study cohort: 97.1% (168) for age, 93.6% (162) for the duration of implantation, and 82.1% (142) for the preoperative clinical diagnosis. Most hips were treated for advanced stages of primary osteoarthritis (71.8%). Other conditions were developmental hip dysplasia (11.3%), femoral head osteonecrosis (7.0%), posttraumatic arthritis (4.9%), and rheumatoid arthritis (4.9%). The remaining 31 cases with unknown patient sex but informative results on the morphological analyses made up the control group (Table 2).

**Table 1:** Cases not included in the present study

reason for not including in the study	men (n) (median age [years], IQR)	women (n) (median age [years], IQR) or (age of years)
bone tissue absent	n = 7	n = 13
(n = 46)	59, 55 to 69	62, 58 to 65
		
bone tissue severely damaged	n = 3	n = 2
(n = 16)	57,56 to 66	53, 53
		
bone tissue sent without fixation	n = 0	n = 5
(n = 11)		56, 40 to 63
		
periprosthetic infection	n = 3	n = 2
(n = 6)	48, 38 to 58	47, 65
		
total	n = 13	n = 22
(n = 79)	57, 54 to 65	59, 53 to 64

**Table 2:** Demographic and clinical characteristics of the study cohort

		entire study cohort	entire study cohort n = 204		valid data on sex n = 173	
			unknown sex n = 31	valid data on sex n = 173	men n = 88	women n = 85
age (median age [years], IQR)		56, 50 to 60	51, 48 to 60	56, 50 to 60	56, 51 to 60	56, 49 to 60
			*P *= 0.531		*P *= 0.584	
clinical diagnosis	osteoarthritis	107	5	102	54	48
	avascular necrosis of femoral head	10	0	10	8	2
	rheumatoid arthritis	7	0	7	3	4
	arthritis secondary to trauma	7	0	7	3	4
	developmental hip dysplasia	17	1	16	6	10
			*P *= 0.867		*P *= 0.288	
THRA design	ASR™	143	18	125	66	59
	DUROM^®^	16	1	15	8	7
	Cormet™	20	6	14	6	8
	BHR™	14	4	10	4	6
	ReCAP^®^	11	2	9	4	5
			*P *= 0.127		*P *= 0.877	
duration of implantation (median in situ time [days], IQR)		147, 51 to 399	127, 25 to 570	147, 55 to 384	124, 54 to 327	182, 55 to 445
			*P *= 0.724		*P *= 0.373	
size of the femoral component (median diameter [mm], IQR)		46, 44 to 50	46, 44 to 50	46, 42 to 48	50, 48 to 52	44, 42 to 46
			*P *= 0.175		*P *= 0.000	

The specimens came from patients with five hip resurfacing femoral systems (Table 2): 125 Articular Surface Replacements (ASR™;DePuy Orthopaedics Inc, Warsaw, IN), 15 DUROM^® ^(Zimmer Inc, Warsaw, IN), 14 Cormet™(Corin Group PLC, Cirencester, UK), 10 Birmingham Hip Resurfacing (BHR™; Smith & Nephew, London, UK), and 9 ReCAP^® ^(Biomet Inc, Warsaw, IN).

All revisions were unilateral. One hundred and fourteen revisions (66%) out of a total of 173 cases with valid data on patient sex were performed for periprosthetic fractures, 45 (26%) for non-fractural causes, and 14 (8%) for acetabular loosening (Table 3). Several cases had more than one reason for revision surgery, for example several hips with pseudoarthrosis hidden under the femoral component caused by chronic fracture were clinically or radiographically classified as loosening of the femoral component.

**Table 3:** The prevalence of distinct failure patterns in the study cohort

		entire study cohort n = 204	entire study cohort n = 204		valid data on sex n = 173	
			unknown sex n = 31	valid data on sex n = 173	men n = 88	women n = 85
cause for revision	periprosthetic fracture	133	19	114	65	49
	non-fractural cause	71	12	59	23	36
			*P *= 0.683		*P *= 0.026	
periprosthetic fracture pattern	postnecrotic biomechanic	73 60	9 10	64 50	41 24	23 26
			*P *= 0.619		*P *= 0.086	
osteonecrosis	present	174	23	151	80	71
	absent	30	8	22	8	14
			*P *= 0.093		*P *= 0.151	
extent of osteonecrosis [mm]		7.4,	6.4,	7.6,	15.3,	6.2,
		2.9 to 19.5	2.1 to 6.8	3.1 to 21.3	3.6 to 24.2	2.6 to 14.6
			*P *= 0.171		*P *= 0.008	
non-fractural causes	Loosening of the acetabular component	15	1	14	4	10
	Loosening of the femoral component	10	3	7	2	5
	cement-socket debonding	3	0	3	2	1
	Collapsed osteonecrosis	5	2	3	0	3
	Metallosis	2	1	1	0	1
	Unexplained groin pain	36	5	31	14	17
			*P *= 0.249		*P *= 0.417	
unexplained groin pain	excessive lymphocyte infiltration	14	3	11	1	10
			*P *= 0.451		*P *= 0.021	
	interface hyperosteoidosis	37	7	30	8	22
			*P *= 0.457		*P *= 0.005	

### Morphological classification of failure patterns

Each specimen was cut using a water-cooled band saw and analyzed macroscopically, contact radiographically and microscopically according to a high standard sampling protocol as described previously [14-18]. Briefly, the femoral heads with *in situ *femoral components were cut in the coronal plane and X-rayed and documented photographically. A second section was oriented perpendicular to the first. The coronal plane and the anterior section were embedded without decalcification in their full length and microscopically analyzed. Each case was examined macroscopically, microscopically and by contact radiography. In our previous work, we proposed classifications for both periprosthetic fractures [18] and the loosening of the femoral component [17] based mostly on the macroscopic and contact radiographic findings which were subsequently confirmed microscopically (for example osteonecrosis, pseudoarthrosis). Histopathological analyses also revealed findings that could not be recognized by macroscopic assessment (for example intraosseous lymphocyte infiltration, hyperosteoidosis of the interface bone trabeculae). We summarized all the results of the histopathological analyses, both macroscopic and microscopic, and proposed classification schemas under the term "morphological patterns" of THRA failure.

**Figure 1:**
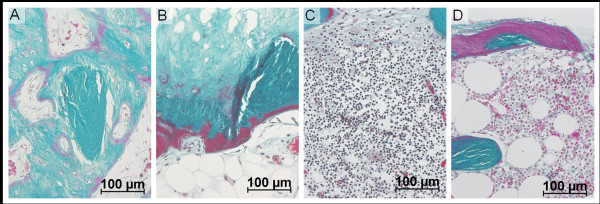
**Morphological findings in retrieved hip resurfacing arthroplasty**. (A) Callus formation in chronic fracture. (B) Osteonecrosis (above) with bordering sclerosis (mid) and adjacent viable fatty bone marrow (lower) distal from the osteonecrotic lesion. (C) Excessive intraosseous lymphocyte infiltration in the vicinity of the bone-cement interface (above). (D) Hyperosteoidosis of bone trabecula at the bone-cement interface (above). (A-D: stain: Goldner trichrome, original magnification: ×200).

Briefly, the periprosthetic fractures were morphologically classified [18] as postnecrotic, when advanced osteonecrosis was found in the complete femoral remnant proximal to the fracture line [14,15], or as biomechanical, when the bone tissue from both sides of the fracture line was proven viable by histopathology. In cases of acute fracture, no reparative reaction was present. In hips with chronic fracture, either the fracture callus (union) or pseudoarthrosis (non-union) was detected microscopically (Figure 1A) [18].

Advanced osteonecrosis was defined macroscopically by yellowish colored areas of the bone and confirmed microscopically by the presence of trabeculae without stainable osteocytes, disorganized bone marrow, and bordering fibrosis (Figure 1B). Because all osteonecrotic lesions showed contact with the surface of the femoral remnant under the prosthesis, we also measured the vertical distance between the bone remnant surface and bordering fibrosis [14].

Excessive intraosseous lymphocyte infiltration was characterized microscopically by the finding of more than 300 lymphocytes within one high power field of the microscope in areas with maximum intraosseous lymphocyte infiltration (Figure 1C) [15].

Interface hyperosteoidosis was defined microscopically by the presence of widened osteoid seams on the trabecular surface at the bone-cement interface. These areas represented compact but somewhat irregular non-mineralized bone tissue within lamellar structured viable superficial bone trabeculae (Figure 1D). These were oriented mostly parallel to the surface of the cement in the vicinity of the cement mantle and also next to intertrabecular cement interdigitations, irrespective of the direction of the intratrabecular lamellae [17].

Failures were defined as clinical complications leading to the revision surgery with loss of the THRA device. One hundred and thirty-three (65%) out of a total of 204 cases provided reproducible results of the morphological analyses showing failure due to periprosthetic fracture. Seventy-one hips were revised for reasons other than the fracture: loosening of the acetabular component (n = 15), loosening of the femoral component (n = 10), cement-socket debonding (n = 3), collapsed osteonecrosis (n = 5), macroscopic visible metallosis (n = 2) and unexplained groin pain (n = 36). Even though several potential causes of the groin pain have been discussed in the literature (for example femoro-acetabular impingement or hypersensitivity reaction), we did not obtain any further specific information and included such cases in the group of 'unexplained groin pain'.

### Statistical methods

Descriptive statistics were performed to describe the median and interquartile range (IQR). As time to revision surgery, the vertical extent of osteonecrosis, and age deviated from a normal distribution, a non-parametric analytical method was used (Mann-Whitney-U test). Logistic regression analysis was used to estimate odds ratios (OR) and 95%- confidence intervals (95% CI). In order to evaluate the possible influence of other variables on the failure pattern of THRA, the size of the femoral component (women commonly need smaller sized prostheses) and clinical diagnoses, logistic regression analysis was also performed after adjustment for sex, age, size of the femoral component, and clinical diagnoses. In the adjusted models, age (in years) and the femoral component size (in millimeters) were used as continuous covariates; for the categorical variables sex and clinical diagnosis all categories were compared to a reference category. We used a global F-test for clinical diagnosis to overcome the problem of sparse subgroups. Although the main focus of our study did not lie in reporting distinct morphological failure patterns for different clinical diagnoses, but instead in investigating the potential cofounders for the examined sex effect, we included these factors in our adjusted models.

## Results

Periprosthetic fracture was the reason for the revision surgery for 65 (73.9%) men and 49 (57.6%; OR: 0.482, 95% CI: 0.254, 0.915; *P *= 0.026) women. The time to failure of male patients (median *in situ *time 85 days, IQR 45 to 185) did not differ significantly from the *in situ *time in women (median *in situ *time 65 days, IQR 33 to 206; *P *= 0.250). Logistic regression analysis with adjustment confirmed the trend for lower ORs for female patients (adjusted OR: 0.290, 95% CI: 0.105, 0.798; *P *= 0.017), but increased ORs for older persons (adjusted OR: 1.049, 95% CI: 1.001, 1.098; *P *= 0.048) with THRA that failed due to periprosthetic fracture (Table 4).

**Table 4:** Periprosthetic fractures in the cohort of retrieved hip resurfacing arthroplasty

	OR	95% CI for OR		Significance
		Lower	Upper	(*P*-value)
Sex (female)	0.482	0.254	0.915	0.026
Size of the Femoral Component	1.031	0.959	1.109	0.408
**Adjusted for Sex, Age, Size of the Femoral Component, and Clinical Diagnosis**				
Sex (female)	0.290	0.105	0.798	0.017
Age	1.049	1.001	1.098	0.048
Size of the Femoral Component	0.948	0.848	1.060	0.349
Clinical Diagnosis				0.060
Osteonecrosis vs. OA^a^	0.154	0.034	0.698	0.015
Rheumatoid Arthritis vs. OA^a^	0.656	0.117	3.684	0.632
Posttraumatic Arthritis vs. OA^a^	4.798	0.481	47.889	0.182
Hip Dysplasia vs. OA^a^	0.568	0.180	1.794	0.335

^a^primary osteoarthritis

Logistic regression analysis and a global F test (for clinical diagnosis).

Osteonecrosis was detected in the femoral remnants of 80 (90.9%) male and 71 (83.5%; OR: 0.507, 95% CI: 0.201, 1.280; *P *= 0.151) female patients. The vertical extent of osteonecrosis was, however, significantly larger in the femoral remnants of male patients (median vertical extent of osteonecrosis 15.3 mm, IQR: 3.6 to 24.2) compared with female patients (median vertical extent of osteonecrosis 6.2 mm, IQR: 2.6 to 14.6; *P *= 0.008). Moreover, 41 (63.1%) out of 65 hip fractures in men were defined as postnecrotic, with a slightly lower frequency in female patients (23 (46.9%) out of 49 periprosthetic fractures were postnecrotic, *P *= 0.086). Interestingly, after adjusting for sex, age, and size of the femoral component, the logistic regression analysis revealed lower ORs for the occurrence of osteonecrosis within the femoral remnants for female patients (adjusted OR: 0.159, 95% CI: 0.040, 0.634; *P *= 0.009) compared with men (Table 5).

**Table 5:** Osteonecrosis in retrieved hip resurfacing arthroplasty

	OR	95% CI for OR		Significance
		Lower	Upper	(*P*-value)
Sex (female)	0.507	0.201	1.280	0.151
Size of the Femoral Component	0.978	0.883	1.084	0.676
**Adjusted for Sex, Age, Size of the Femoral Component, and Clinical Diagnosis**				
Sex (female)	0.159	0.040	0.634	0.009
Age	1.010	0.952	1.072	0.738
Size of the Femoral Component	0.867	0.748	1.004	0.057
Clinical Diagnosis				0.579
Osteonecrosis vs. OA^a^	0.255	0.050	1.295	0.099
Rheumatoid Arthritis vs. OA^a^	1.352	0.129	14.135	0.801
Posttraumatic Arthritis vs. OA^a^	n.e.^b^	-	-	-
Hip Dysplasia vs. OA^a^	n.e^.b^	-	-	-

^a^primary osteoarthritis; ^b^not estimable

Logistic regression analysis and a global F test (for clinical diagnosis)

Excessive intraosseous lymphocyte infiltration of femoral remnant bone tissue was observed in 11 (6.4%) hips. Ten patients with unexplained groin pain and excessive lymphocyte infiltration of the femoral remnant were women (OR: 11.600, 95% CI: 1.451, 92,731; *P *= 0.021). In addition, larger femoral components had a lower OR (OR: 0.810, 95% CI: 0.689, 0.953; *P *= 0.011). After adjusting the analysis, a similar strong correlation was detected for excessive lymphocyte infiltration in women (adjusted OR: 10.216, 95%CI: 0.787, 132.574; *P *= 0.076), but not for the size of the femoral component (adjusted OR: 0.971, 95% CI: 0.779, 1.210; *P *= 0.792; Table 6).

**Table 6:** Excessive lymphocyte infiltration of bone remnant tissue in retrieved hip resurfacing arthroplasty

	OR	95% CI for OR		Significance
		Lower	Upper	(*P*-value)
Sex (female)	11.600	1.451	92.731	0.021
Size of the Femoral Component	0.810	0.689	0.953	0.011
**Adjusted for Sex, Age, Size of the Femoral Component, and Clinical Diagnosis**				
Sex (female)	10.216	0.787	132.574	0.076
Age	0.969	0.891	1.055	0.471
Size of the Femoral Component	0.971	0.779	1.210	0.792
Clinical Diagnosis				0.886
Osteonecrosis vs. OA^a^	3.546	0.288	43.597	0.323
Rheumatoid Arthritis vs. OA^a^	1.982	0.161	24.437	0.593
Posttraumatic Arthritis vs. OA^a^	n.e.^b^	-	-	-
Hip Dysplasia vs. OA^a^	n.e.^b^	-	-	-

^a^primary osteoarthritis; ^b^not estimable

Logistic regression analysis and a global F test (for clinical diagnosis).

Hyperosteoidosis at the bone-cement interface was observed in 30 (17.3%) out of all 173 cases. Of these, 22 (73.3%) were women (OR: 3.492, 95% CI: 1.457, 8.368; *P *= 0.005). The relationship between interface hyperosteoidosis and the size of the femoral component was not significant (OR: 0.918, 95% CI: 0.836, 1.009; *P *= 0.076). After adjusting the analysis for sex, age, the size of the femoral component and clinical diagnosis, the interface hyperosteoidosis showed a strong association with female sex (adjusted OR: 4.190, 95% CI: 1.142, 15.376; *P *= 0.031; Table 7).

**Table 7:** Hyperosteoidosis of the bone trabeculae at the bone-cement interface in retrieved hip resurfacing arthroplasty.

	OR	95% CI for OR		Significance
		Lower	Upper	(*P*-value)
Sex (female)	3.492	1.457	8.368	0.005
Size of the Femoral Component	0.918	0.836	1.009	0.076
**Adjusted for Sex, Age, Size of the Femoral Component, and Clinical Diagnosis**				
Sex (female)	4.190	1.142	15.376	0.031
Age	0.496	0.928	1.037	0.471
Size of the Femoral Component	1.003	0.876	1.037	0.961
Clinical Diagnosis				0.733
Osteonecrosis vs. OA^a^	2.909	0.586	14.430	0.191
Rheumatoid Arthritis vs. OA^a^	n.e.^b^	-	-	-
Posttraumatic Arthritis vs. OA^a^	n.e.^b^	-	-	-
Hip Dysplasia vs. OA^a^	0.771	0.180	3.312	0.727

^a^primary osteoarthritis; ^b^not estimable

Logistic regression analysis and a global F test (for clinical diagnosis).

## Discussion

### Summary of main findings

We investigated the possible sex differences in failure patterns of the current generation of metal-on-metal hip resurfacing arthroplasty. We analyzed morphologically distinct failure modes in a large collection of retrieved hips and performed statistical analyses. We observed substantial sex differences in the failure patterns of hip resurfacing arthroplasty: male hips showed more frequent osteonecrosis with larger lesions than those of women and osteonecrosis led to fracture more frequently in men. On the other hand, the bone remnants of women were more likely to contain excessive lymphocyte infiltrations and to show interface hyperosteoidosis, both of which were linked to unexplained persistent groin pain associated with suggested hypersensitivity reaction.

### Explaining the results and comparing them with those of other studies

Following improvements in metallurgy and surgical technique, patient selection remains an important tool with which to positively influence the outcome of metal-on-metal hip arthroplasty. Although men under the age of 65 with osteoarthritis are considered to be the best candidates for hip resurfacing based on data from registries and larger centers [2-6,8-12], such data are mostly relatively unstructured and do not provide an adequate answer to the question, how do other factors such as the diameter of the prosthesis, age of the patient or clinical diagnosis influence the prosthesis failure? Recently, McBryde and associates computed a multivariate Cox proportional hazard survival model, and found that increased risk was related to differences in the size of the femoral component in their cohort of 48 failures (out of a total of 2,123 implanted hips) [13]. Similarly, in their study cohort of 1,107 resurfaced hips, Amstutz and collaborators reported a higher revision rate in women, although the effect of sex disappeared after adjustment for component size and surgical technique [7]. In contrast to clinical studies, we analyzed a large cohort of standardly analyzed retrieved hip resurfacing arthroplasties and focused on several morphologically well-defined lesions within the remnant tissue that had previously been suggested to show some degree of sexual dimorphism. It seems likely that further classification of characteristic failure modes into subgroups will enable further insight into the different biological reactions to prostheses in men and women. Similarly to our results [14], Little and colleagues [19] also found osteonecrosis in the majority of fractures in male patients. Moreover, the suggestion that female patients suffer from hypersensitivity reactions to prostheses more frequently than males is generally accepted [15,17,20,21].

### Limitations of the study

We recognize several important limitations to the present study. First, we were unable to estimate the total population of patients with implanted THRA operated on by the cooperating surgeons, and therefore the prevalence, preoperative and postoperative functional scorings and other possible risk factors remain unknown. Moreover, we cannot exclude that some surgeons did not send all their retrieved hips to our laboratory or that some revision surgeries were possibly performed by other than our cooperating surgeons (selection bias). However, to reduce further selection bias, all cases with informative morphological findings were included in the current study and we also present our complete data on all specimens submitted to the Hamburg retrieval study on hip resurfacing arthroplasty. Furthermore, as only 8 to 15 failures were obtained for four of the five studied designs, we did not further differentiate between the different designs of prostheses. However, it must be noted that in our study cohort, association of THRA design with distinct failure modes was not observed. To minimize classification bias, all specimens were processed according to highly standard schema and we did histological analysis from three quadrants from each retrieved hip. In our previous work, we also investigated inter- and intra-observer agreement for qualitative diagnoses such as the presence of osteonecrosis [14,18] and the final diagnoses were assigned by consensus between two investigators (MA, JZ). In terms of the morphological changes associated with the potential delayed-type hypersensitivity reaction, it should be kept in mind that there is no consensus about the specific histopathological features of this complication. While some investigators suggested that anterior solid granulomatous pseudotumors [20,21] are specific for hypersensitivity, newer data observed malpositioning leading to the accumulation of metal wear particles directly within such lesions [22-24]. In the few cases in our study cohort that seemed to be associated with metal hypersensitivity, we observed proliferative desquamative synovitis linked with joint effusion under pressure and excessive intraosseous lymphocyte infiltration [15]. Because confidence intervals for some categories were quite wide, a reclassification of a single case (for example in a group of 11 cases showing excessive lymphocyte infiltration) may possibly change these substantially. To overcome the problem of inter-observer variability in semiquantitative diagnoses (for example moderate versus severe lymphocyte infiltration), we therefore defined intraosseous excessive lymphocyte infiltration quantitatively as more than 300 cells in one high power field [15], which represented a very conservative cutoff value. We also reported interface hyperosteoidosis [17] occurring preferentially in failures in female patients, but its possible association with the hypersensitivity reaction remains unclear until specific tests for metal allergy are available.

### Implications for research and clinical practice

In the current study, we demonstrated that detailed classification of distinct failure patterns of prostheses might help to explain the differing pathogenesis of such complications and enable future stratification of risk factors as well as different therapeutic strategies for certain patient populations (gender medicine and/or personalized medicine). Specific diagnostics of (as minimally invasive as possible) and therapy for (immunomodulatory instead of operative) the hypersensitivity reaction to prostheses remains an important issue for future interdisciplinary research in orthopedics.

## Conclusions

Within the limitations of this study, we can conclude that, we demonstrated a substantial sex difference in distinct failure patterns of metal-on-metal hip resurfacing. The recognition of pathogenically distinct failure modes will enable further stratification of risk factors for certain failure mechanisms and will influence future therapeutic options for selected patient groups.

## Abbreviations

CI: confidence interval; IQR: interquartile range; OR: odds ratio; THRA: total hip resurfacing arthroplasty.

## Competing interests

The authors declare that they have no competing interests.

## Authors' contributions

JZ is the lead investigator of the study and developed its design, carried out data acquisition, and supervised data analysis and interpretation. AH and EV carried out data analysis and interpretation and helped to prepare the manuscript. MM, WR and MA provided important intellectual contributions to the study and manuscript preparation. All authors read, edited and approved the final manuscript.

## Note

The study was supported by DePuy Orthopaedics, Inc, Warsaw IN (MA, MM); Smith&Nephew, London, UK (MA, MM); Corin Group PLC, Cirencester, UK (MA, MM); Zimmer Inc, Warsaw, IN (MA, MM); and Biomet Inc, Warsaw, IN (MA, MM).

## Pre-publication history

The pre-publication history for this paper can be accessed here:

http://www.biomedcentral.com/1741-7015/9/113/prepub
